# Cartilage boundary lubrication synergism is mediated by hyaluronan concentration and PRG4 concentration and structure

**DOI:** 10.1186/s12891-015-0842-5

**Published:** 2015-12-14

**Authors:** Taryn E. Ludwig, Miles M. Hunter, Tannin A. Schmidt

**Affiliations:** Biomedical Engineering Graduate Program, University of Calgary, Calgary, Canada; Faculty of Kinesiology, University of Calgary, Calgary, Canada

**Keywords:** Cartilage, Hyaluronan, Proteoglycan 4, Boundary Lubrication

## Abstract

**Background:**

Proteoglycan 4 (PRG4) and hyaluronan (HA) are key synovial fluid constituents that contribute synergistically to cartilage boundary lubrication; however, the effects of their concentrations as well as their structure, both of which can be altered in osteoarthritis, on this functional synergism are unknown. The objectives of this study were to evaluate cartilage boundary lubricating ability of 1) PRG4 + HA in solution at constant HA concentration in a range of PRG4 concentrations, 2) constant PRG4 concentration in a range of HA concentrations, 3) HA + reduced/alkylated (R/A) PRG4, and 4) hylan G-F 20 + PRG4.

**Methods:**

Static and kinetic friction coefficients (μ_static,Neq_, <μ_kinetic,Neq_>) were measured using a previously characterized cartilage-cartilage boundary mode friction test for the following concentrations of purified PRG4 and HA: Test 1: HA (1.5 MDa, 3.3 mg/mL) + PRG4 from 4.5 – 1500 μg/mL; Test 2: PRG4 (450, 150, 45 μg/mL) + HA (1.5 MDa) from 0.3 – 3.3 mg/mL. Test 3: hylan G-F 20 (3. 3 mg/mL) + PRG4 (450 μg/mL). Test 4: HA (3.3 mg/mL) + R/A PRG4 (450 μg/mL). ANOVA was used to compare lubricants within (comparing 6 lubricants of interest) and between (comparing 3 lubricants of interest) test sequences, with Tukey and Fishers post-hoc testing respectively.

**Results:**

This study demonstrates that both PRG4 and HA concentration, as well as PRG4 disulfide-bonded structure, can alter the cartilage boundary lubricating ability of PRG4 + HA solutions. The boundary lubricating ability of high MW HA + PRG4 solutions was limited by very low concentrations of PRG4. Decreased concentrations of high MW HA also limited the cartilage boundary lubricating ability of HA + PRG4 solutions, with the effect exacerbated by low PRG4 concentrations. The reduction of friction by addition of PRG4 to a cross-linked HA viscosupplement product, but not with addition of R/A PRG4 to HA, is consistent with a non-covalent mechanism of interaction where tertiary and quaternary PRG4 structure are important.

**Conclusions:**

Collectively, these results demonstrate that deficiency of either or both PRG4 and HA, or alterations in PRG4 structure, may be detrimental to SF cartilage boundary lubricating function. This study provides further insight into the nature of cartilage boundary lubrication and advancement towards potential formulation of new intra-articular biotherapeutic treatments for osteoarthritis using PRG4 ± HA.

## Background

Friction between articular cartilage surfaces in motion is mediated through a combination of lubrication mechanisms. During fluid film lubrication, cartilage surfaces are separated by a fluid layer, while during boundary lubrication friction is mediated by interactions between lubricant molecules adsorbed to the surface [1]. The boundary lubrication mode becomes increasingly dominant as loading time is increased and interstitial fluid is depressurized [2, 3]. Furthermore, opposing cartilage surfaces make contact over only approximately 10 % of the total area, making these areas of contact vulnerable to high friction [4]. Synovial fluid (SF) constituents proteoglycan 4 (PRG4) and hyaluronan (HA) are the primary contributors to its cartilage boundary lubricating ability [5]. PRG4 [6] is a mucin-like O-linked glycosylated protein present in SF [7] and at the articular cartilage surface [8]. HA, a linear polymer of D-glucuronic acid and D-N-acetylglucosamine [9], is also present in SF. Alone, solutions of PRG4 or HA reduce friction in a dose-dependent manner at a cartilage-cartilage biointerface in a boundary mode of lubrication compared to phosphate buffered saline (PBS). When combined at physiological concentrations, PRG4 + HA further reduce friction synergistically towards that of whole SF. [5] Both PRG4 and HA are critical to the cartilage boundary lubricating function of SF, and decreased boundary lubricating ability of SF has been linked with increased wear at the articular surface [10].

While the molecular mechanism of the PRG4 + HA synergism at a cartilage-cartilage biointerface in a boundary mode of lubrication remains to be fully understood, some characterization of potential factors affecting the synergism *in vitro* has previously been performed. In solutions of HA alone, friction coefficients decrease with increasing HA concentration [5, 11], and slightly with increasing molecular weight (MW, from 20 kDa to 5 MDa, at a concentration of 3.3 mg/ml) [11, 12]. However, upon addition of PRG4 at 450 μg/mL the dependence of friction coefficient on HA MW is no longer observed [12] and friction is reduced to a similar value by addition of PRG4 over the range of MW of the 3.3 mg/ml HA solutions. Some SF from patients with osteoarthritis (OA) is deficient in PRG4, has normal HA concentration, an HA MW distribution shifted towards the lower range over all sizes from 6 MDa to 0.5 MDa, and fails to lubricate as well as normal SF. Normal cartilage boundary lubricating ability could be restored with addition of PRG4 to the SF [13], as evidenced by a measured reduction in friction. A similar decrease in SF HA concentration and HA MW, although with an increase in PRG4 concentration, has been observed in an equine acute injury model; this SF also fails to lubricate, though cartilage boundary lubricating ability could be restored by supplementation with high MW HA (4 MDa), but not low MW HA (800 kDa) [11]. These studies collectively demonstrate that both PRG4 and HA, particularly high MW HA, are necessary contributors to the cartilage boundary lubricating function of SF. However, the potential concentration dependence of PRG4 and/or high MW HA, both of which can be diminished in diseased SF, of the functional friction-reducing PRG4 + HA synergism at a cartilage-cartilage biointerface remains to be fully clarified.

The effects of injury and disease on PRG4 structure in SF, including relative composition of multimers:monomers and fragments of PRG4 [14], remain to be fully elucidated. As PRG4 is known to be degraded by enzymes such as neutrophil elastase, which can be up-regulated in inflammatory conditions such as post-anterior cruciate ligament tear [15], the ability of its fragments to maintain their ability to interact with HA may be of functional significance. The lubricating ability of PRG4 is decreased after it is reduced and alkylated (R/A) to break both inter- and intra-molecular disulfide bonds [16], and preparations of PRG4 enriched in disulfide-bonded multimeric species provide enhanced lubricating ability compared to preparations enriched in monomeric PRG4 [17]; this demonstrates the functional importance of inter-molecular disulfide bonds specifically, as reduced preparations of monomers appear to lubricate as well as non-reduced monomers [18]. Furthermore, R/A decreases the ability of PRG4 to adsorb to cartilage surfaces [19]. However, the effect of loss of disulfide-bonded structure, which may occur in diseased SF, by R/A on PRG4’s ability to interact with HA and synergistically reduce friction in a boundary mode at a cartilage-cartilage biointerface is also unknown.

Lastly, the MW of HA has also been linked to its efficacy as an intra-articular viscosupplement. Intra-articular HA injections are currently used to treat pain in OA patients, and it is thought that increasing the MW of HA by cross-linking increases joint residence time [20]. Increased MW may also contribute to pain relief by increased protection of nerve endings via increased viscosity [21]. Hylan G-F 20 (“Synvisc”, Genzyme) is one such example of a cross-linked HA preparation currently available and used clinically for intra-articular injections [22]. Given the clinical utility of cross-linked HA preparations, and evidence for intra-articular administration of PRG4’s potential efficacy in preventing joint degradation in animal models of OA [23–26], the ability of cross-linked HA to functionally interact with PRG4 synergistically to reduce friction in a boundary mode at a cartilage biointerface, towards that of whole SF, is of significant interest and currently unknown.

Given the limited level of understanding pertaining to the concentration and structural dependency of PRG4 + HA synergistic cartilage boundary lubrication function, and the clinical correlations of SF cartilage boundary lubricant composition and function to joint health and disease, the objectives of this study were as follows: to evaluate cartilage boundary lubricating ability of 1) PRG4 + HA in solution at constant HA concentration in a range of PRG4 concentrations, 2) constant PRG4 concentration in a range of HA concentrations, 3) HA + R/A PRG4, and 4) hylan G-F 20 + PRG4.

## Methods

### Materials

Materials for lubrication testing were obtained as described previously [12]. HA of 1.5 MDa MW was obtained from Lifecore Biomedical LLC (Chaska, MN, USA), and bovine SF was obtained from Animal Technologies (Tyler, TX, USA). Skeletally mature bovine stifle joints (equivalent to a human knee joint) were obtained from a local slaughterhouse (Calgary, AB, Canada) under approval by the Animal Care Committee at the University of Calgary. Hylan G-F 20 was from Sanofi Canada (Laval, QC, Canada). PRG4 was purified from culture media conditioned by mature bovine cartilage explants, as described previously [5]. Purity of the PRG4 preparation was confirmed by 3–8 % Tris-Acetate SDS-PAGE followed by protein stain and Western blotting with anti-PRG4 antibody 5C11 (obtained from Millipore, Etobicoke, ON, Canada) [27] with Invitrogen’s NuPAGE system. Concentration of the purified PRG4 was determined by bicinchoninic acid assay.

**Fig. 1:**
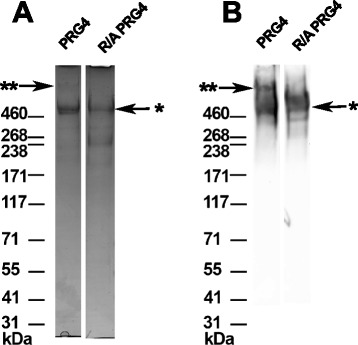
Characterization of PRG4 and reduced and alkylated (R/A) PRG4. Protein stain on 3-8 % Tris-Acetate SDS-PAGE stained with SimplyBlue SafeStain (**a**) and immunoreactivity with anti-PRG4 antibody 5C11 (**b**). ** and * indicate high MW multimeric species and monomeric PRG4 species, respectively, with the former being present in the non-reduced samples and absent from the R/A PRG4

Lubricants were prepared by combining the required volumes of PRG4 (prepared in PBS) and HA (prepared in PBS) at the appropriate concentrations. Hylan G-F 20 (initially 8.0 mg/mL) was diluted to 3.3 mg/mL in PBS. R/A PRG4 was prepared in PBS by incubation with 10 mM dithiothreitol for 2 h at 60 °C and then 40 mM sodium iodoacetate for 2 h at room temperature in the dark [27], followed by dialysis against PBS overnight at 37 °C. R/A was confirmed by SDS-PAGE followed by protein staining [28]. Figure 1 demonstrates the purity of the PRG4 preparation as well as confirmation of R/A of PRG4.

### Sample preparation

Annulus and core shaped osteochondral samples (*N* = 42 pairs) were harvested from the patellofemoral groove of 11 skeletally mature bovine stifle joints as described previously [29]. An annular contact area was used to reduce variations in speed across the cartilage surfaces. Samples were rinsed vigorously overnight in ~40 mL of PBS at 4 °C to remove residual SF from the cartilage surface. Samples were then stored at −80 °C in PBS with protease inhibitors until the day prior to testing, at which time they were thawed and again rinsed vigorously overnight in PBS. Samples were then bathed in the next day’s test lubricant (0.2 mL for core, 0.1 mL for annulus), such that the cartilage surface was completely immersed, at 4 °C overnight prior to lubrication testing.

### Lubrication testing

Lubrication tests were performed on a Bose ELF 3200 using a previously characterized in vitro cartilage-on-cartilage boundary mode lubrication test. [29] Briefly, annulus and core shaped osteochondral samples were opposed against each other, resulting in a stationary contact area, compressed to 18 % of the total cartilage thickness at 0.002 mm/s, and an interstitial fluid depressurization period of 40 minutes was allowed resulting in ~0.09 MPa compressive stress. Without removal of this equilibrium load (N_eq_) samples were then left opposed under N_eq_ for pre-sliding durations (Tps) of 1200, 120, 12, and 1.2 s, and rotated +2 revolutions and −2 revolutions at 0.3 mm/s after each Tps. This test sequence was then repeated in the opposite direction of rotation. Using the N_eq_, static (μ_static,Neq_), and kinetic (<μ_kinetic,Neq_>) coefficients of friction were calculated [29], representing the resistance to the onset of motion and steady motion, respectively. Because < μ_kinetic,Neq_ > increased only slightly with Tps, with values at Tps = 1.2 s being on average within ~18 % of those at Tps = 1200 s, for brevity and clarity, and as done previously [5], only < μ_kinetic,Neq_ > at Tps = 1.2 s will be presented.

In all experiments, each osteochondral pair was tested sequentially over 4–5 days in each of the 4–5 test lubricants. Lubricants were selected in order of predicted increasing lubricating ability to minimize carryover effects. In all tests, PBS served as the negative control lubricant and bovine SF served as the positive control lubricant. PRG4 and HA concentrations were selected to represent values lower, similar, and higher, to those observed in normal human SF [13].

### Lubricant sequences

Four sets of tests were performed to evaluate cartilage boundary lubricating ability of varying concentrations of PRG4 and HA, as well that of hylan G-F 20 ± PRG4 and HA + R/A PRG4. Lubricant sequences for each test are shown in Table 1. To determine the effect of PRG4 concentration on PRG4 + HA cartilage boundary lubricating ability in a constant [HA] = 3.3 mg/mL, a high dose of PRG4 (Test 1A , 150 – 1500 μg/mL, *N* = 6) and a low dose of PRG4 (Test 1B, 4.5 – 150 μg/mL, *N* = 4) were performed. Test sequences 1A and 1B were pooled for analysis. To determine the effect of HA concentration on PRG4 + HA cartilage boundary lubricating ability in constant [PRG4] three test sequences using HA concentrations ranging from 0.3 – 3.3 mg/mL were performed in PRG4 concentrations of 450 (Test 2A, *N* = 8), 150 (Test 2B, *N* = 4) and 45 μg/mL (Test 2C, *N* = 4). Results were compared between the 3 test sequences at each HA concentration. The cartilage boundary lubricating ability of HA combined with R/A PRG4 (disruption of tertiary and quaternary structure, inter- and intra-molecular disulfide bonds are broken), was evaluated compared to the lubricating ability of HA alone and HA with non-reduced PRG4 (Test 3, *N* = 8). To determine the cartilage boundary lubricating ability of PRG4 combined with cross-linked HA, hylan G-F 20 was tested alone and then in combination with PRG4 at 450 μg/mL (Test 4, *N* = 8).

**Table 1:** Summary of lubricant sequences used over 4 – 5 consecutive days of testing

Test	Lubricant [PRG4] (μg/mL), [HA] (mg/ml)				
	1	2	3	4	5
*1A: PRG4 low dose, HA (N = 4)*	PBS	[PRG4] = 4.5	[PRG4] = 45	[PRG4] = 150	SF
		[HA] = 3.33	[HA] = 3.33	[HA] = 3.33	
*1B: PRG4 high dose, HA (N = 6)*	PBS	[PRG4] = 150	[PRG4] = 450	[PRG4] = 1500	SF
		[HA] = 3.33	[HA] = 3.33	[HA] = 3.33	
*2A: PRG4 high, HA dose (N = 8)*	PBS	[PRG4] = 450	[PRG4] = 450	[PRG4] = 450	SF
		[HA] = 0.3	[HA] = 1.0	[HA] = 3.33	
*2B: PRG4 mid, HA dose (N = 4)*	PBS	[PRG4] = 150	[PRG4] = 150	[PRG4] = 150	SF
		[HA] = 0.3	[HA] = 1.0	[HA] = 3.33	
*2C: PRG4 low, HA dose (N = 4)*	PBS	[PRG4] = 45	[PRG4] = 45	[PRG4] = 45	SF
		[HA] = 0.3	[HA] = 1.0	[HA] = 3.33	
*3: R/A PRG4, HA (N = 8)*	PBS	[PRG4] = 0	R/A [PRG4] = 450	[PRG4] = 450	SF
		[HA] = 3.33	[HA] = 3.33	[HA] = 3.33	
*4: PRG4, cross-linked HA (N = 8)*	PBS	[PRG4] = 0	[PRG4] = 450	SF	N/A
		[HYLAN G-F 20] = 3.33	[HYLAN G-F 20] = 3.33		

### Statistical analysis

Unless otherwise indicated, data are presented as mean ± 95 % confidence interval (upper limit, lower limit). Data was tested for normality using Shapiro-Wilks test and log-transformed if not normal. Non-parametric methods (Wilcoxon signed rank test) were used if data were not normal or normalized with log-transformation. The effects of test lubricant and Tps (as a repeated factor) on friction coefficients, μ_static,Neq_ and < μ_kinetic,Neq_>, were assessed by repeated measures analysis of variance (ANOVA). To compare lubricants within test sequences, the effect of test lubricant on < μ_kinetic,Neq_ > at Tps = 1.2 s between test lubricants and SF was assessed by ANOVA, with Tukey post-hoc testing between the 5 lubricants of interest. To compare lubricants between test sequences (i.e. between the HA dose responses in the three PRG4 concentration sequences), the effect of test lubricant on < μ_kinetic,Neq_ > at Tps = 1.2 s was assessed by ANOVA with protected Fishers post-hoc testing between the 3 lubricants of interest. Statistical analysis was performed using SPSS 22.0 (IBM SPSS software, New York, NY).

## Results

### Lubrication Testing

#### PRG4 dose response in HA

**Fig. 2:**
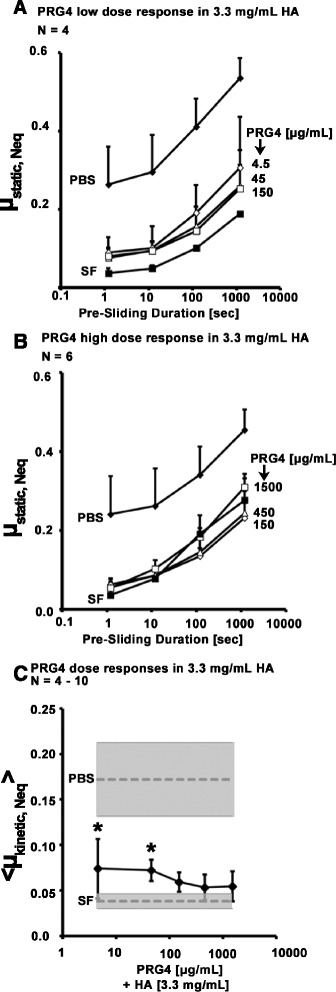
Effect of PRG4 concentration on cartilage boundary lubricating ability. μ_static,Neq_ for PRG4 low dose (Test 1A, **a**), and PRG4 high dose (Test 1B, **b**). <μ_kinetic,Neq_ > at Tps = 1.2 s (**c**) for PRG4 high and low dose response + constant [HA] = 3.3 mg/mL (Tests 1A, 1B). Average < μ_kinetic,Neq_ > in PBS and SF shown in grey for reference. * = significantly higher than SF (*p* < 0.05)

In constant [HA] = 3.3 mg/mL, coefficients of friction appeared to decrease towards that of SF as [PRG4] increased, decreasing towards a plateau between 45 and 150 μg/mL. μ_static,Neq_ varied with test lubricant and Tps in both the low dose sequence (*p* < 0.001, *p* < 0.05, Fig. 2a) and high dose sequence (*p* < 0.001, *p* < 0.001, with an interaction between the effects of lubricant and Tps on μ_static,Neq_ (*p* < 0.01), Fig. 2b). <μ_kinetic,Neq_ > at Tps = 1.2 seconds also varied with test lubricant (*p* = 0.02). Values of < μ_kinetic,Neq_ > in [PRG4] = 4.5 and 45 μg/mL were significantly higher than those in SF (*p* = 0.03, 0.04). <μ_kinetic,Neq_ > in [PRG4] = 150, 450, and 1500 μg/mL were similar to each other and to SF (*p* = 0.14 – 1.0, Fig. 2c).

#### HA dose response in PRG4

**Fig. 3:**
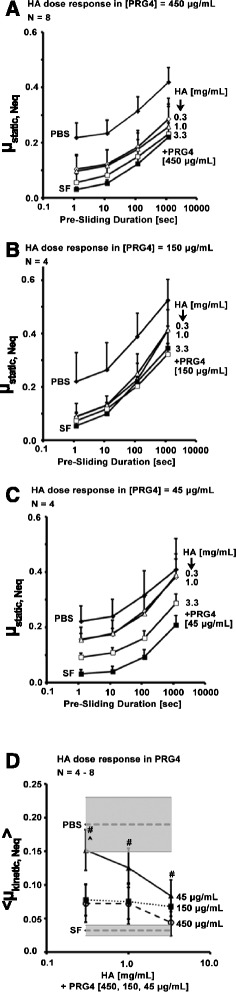
Effect of HA concentration on cartilage boundary lubricating ability. μ_static,Neq_ (**a, b, c**) for HA dose responses + constant [PRG4] = 45 μg/mL (Test 2A, **a**), 150 μg/mL (Test 2B, **b**), and 450 μg/mL (Test 2C, **c**). <μ_kinetic,Neq_ > at Tps = 1.2 s (**d**) for all doses of HA in [PRG4] = 45, 150, 450 μg/mL (Test 2A, 2B, 2C). Average < μ_kinetic,Neq_ > in PBS and SF shown in grey for reference. # = significantly higher than [PRG4] = 450 μg/mL (*p* < 0.05). ^ = significantly higher than [PRG4] = 150 μg/mL (*p* < 0.05)

In [PRG4] = 450 μg/mL, μ_static,Neq_ varied with test lubricant and Tps (all *p* <0.001, Fig. 3a). In [PRG4] = 150 μg/mL, μ_static,Neq_ varied with test lubricant and Tps (*p* = 0.005, *p* < 0.0001) without an interaction (*p* = 0.92, Fig. 3b). In [PRG4] = 45 μg/mL, μ_static,Neq_ also varied with test lubricant and Tps (*p* = 0.001 and *p* < 0.0001) without an interaction (*p* = 0.37, Fig. 3c).

When compared between test sequences, values of < μ_kinetic,Neq_ > were higher in [PRG4] = 45 μg/mL compared to those in [PRG4] = 450 μg/mL at [HA] = 0.3, 1.0 , and 3.3 mg/mL (*p* = 0.002, 0.03, 0.03 respectively, Fig. 3d). At [HA] = 0.3 mg/mL, [PRG4] = 45 μg/mL was also higher than 150 μg/mL (*p* = 0.007). There was no difference between < μ_kinetic,Neq_ > for [PRG4] = 150 and 450 μg/mL at [HA] = 0.3 or 1.0 mg/mL (*p* = 0.82, 0.91), however the difference was appreciable (though not significant) at [HA] = 3.3 mg/mL (*p* = 0.19).

#### R/A PRG4

**Fig. 4:**
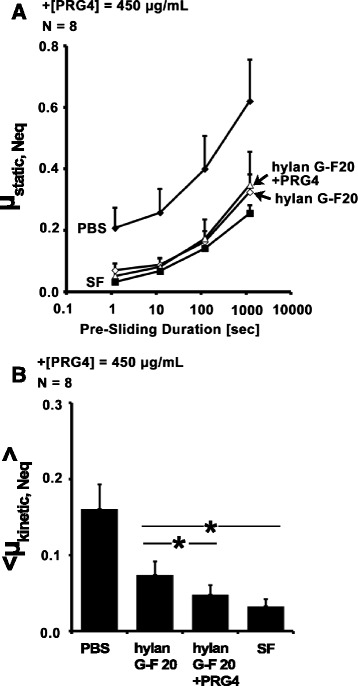
Effect of addition of R/A PRG4 on HA cartilage boundary lubricating ability. μ_static,Neq_ (**a**), and <μ_kinetic,Neq_> at Tps = 1.2 s (**b**) for HA, HA + [R/A PRG4] = 450 μg/mL, and HA + [PRG4] = 450 μg/mL (Test 4). * = p < 0.05

Addition of R/A PRG4 at 450 μg/mL to 1.5 MDa HA at 3.3 mg/mL appeared to slightly, but not significantly, lower friction compared to HA alone. μ_static,Neq_ varied with test lubricant and Tps (all *p* < 0.001, Fig. 4a). <μ_kinetic,Neq_ > at Tps = 1.2 s also varied with test lubricant (*p* < 0.0001, Fig. 4b). <μ_kinetic,Neq_ > for HA alone was significantly higher than SF (*p* = 0.001). Addition of R/A PRG4 to HA did not significantly reduce < μ_kinetic,Neq_ > compared to HA alone (*p* = 0.46), however < μ_kinetic,Neq_ > for HA + R/A PRG4 was significantly higher than SF (*p* = 0.04). Addition of PRG4 to HA tended to improve lubricating ability compared to HA alone (*p* = 0.06), and there were no significant differences between HA + PRG4 and HA + R/A PRG4 or SF (*p* = 0.65, 0.33).

#### Partially cross-linked HA

**Fig. 5:**
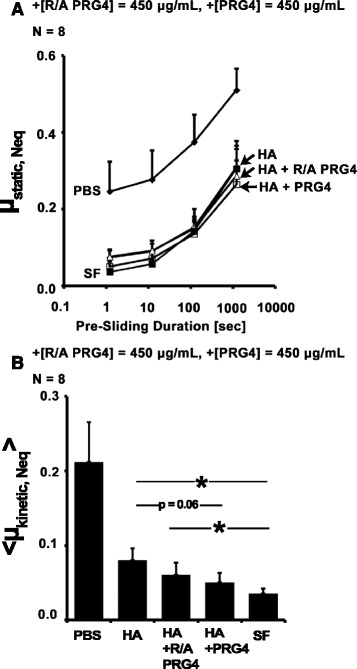
Effect of addition of hylan G-F20 on PRG4 cartilage boundary lubricating ability. μ_static,Neq_ (**a**), and <μ_kinetic,Neq_> at Tps = 1.2 s (**b**) for hylan G-F20 ± [PRG4] = 450 μg/mL (Test 3). * = p < 0.05

Addition of PRG4 at 450 μg/mL to hylan G-F 20 at 3.3 mg/mL decreased friction compared to hylan G-F 20 alone. μ_static,Neq_ varied with test lubricant and Tps with an interaction (all *p* < 0.0001, Fig. 5a). <μ_kinetic,Neq_ > at Tps = 1.2 s also varied with test lubricant (*p* = 0.02, Fig. 5b). Hylan G-F 20 alone failed to lubricate as well as SF (*p* = 0.001). Hylan G-F 20 + PRG4 was significantly lower than hylan G-F 20 alone (*p* = 0.04), and provided boundary lubricating ability equivalent to that of SF (*p* = 0.29).

## Discussion

The results described here demonstrate that concentration of both PRG4 and high MW HA can affect the ability of PRG4 + HA solutions to reduce friction in the boundary mode at a cartilage-cartilage biointerface. The lubricating ability provided by the PRG4 + HA solutions tested here approached that of whole SF except for very low PRG4 (4.5, 45 μg/mL) concentrations in physiologically normal HA concentrations. This diminished cartilage boundary lubricating ability was enhanced when low PRG4 concentrations (45, 150 μg/mL) were added to low HA concentrations (0.3, 1.0 mg/mL); in this case physiological levels of PRG4 reduced friction, but not to the same level as when combined with higher HA concentrations. These results demonstrate that both PRG4 and high MW HA concentration can be limiting in achieving reduction of friction in the boundary mode at a cartilage-cartilage biointerface, and that both are necessary contributors to the cartilage boundary lubricating ability of SF. Furthermore, the addition of R/A PRG4 to HA was unable to significantly reduce friction, indicating that PRG4’s tertiary and quaternary protein structure is important in its friction reducing synergism with HA at a cartilage-cartilage biointerface. Lastly, PRG4 + hylan G-F 20 demonstrated improved lubricating ability compared to hylan G-F 20 alone, indicating that the HA + PRG4 cartilage boundary lubrication synergism is also maintained with a clinically relevant preparation of cross-linked HA. Collectively, these results demonstrate that both PRG4 and HA are necessary for effective friction reduction towards the level of whole SF and suggest that deficiency of either or both may be detrimental to SF cartilage boundary lubricating function.

The in vitro friction test used here is able to quantify contributions of PRG4 and HA to friction reduction in the boundary mode at a physiologically relevant cartilage-cartilage biointerface. The test geometry, protocol, and physiological surfaces allow for friction in a boundary mode of lubrication to be measured, even in viscous HA solutions - as indicated by the observation that PRG4 is able to reduce friction in a dose dependent manner in high MW HA solutions (3.3 mg/mL). While traditional Stribeck curve analysis, originally developed for steel surfaces, is not possible here given the rotational test geometry that facilitates the depressurized, stationary area of contact, its application to biointerfaces composed of porous, hydrogels [30] (e.g. cartilage) has recently been demonstrated to be not appropriate for biological tissues; it is not able to account for the macromolecules present at the deformable cartilage surfaces and in the non-Newtonian lubricant solutions that contribute to friction forces [31]. Though model surfaces provide the advantage of well-defined sample surfaces and modes of lubrication, and have been used to study wear prevention (previous studies have shown that friction and wear are linked at the articular surface [10]) as well as the order in which PRG4 and HA are adsorbed to surfaces [32], they may not allow for all the operative physiological interactions at a cartilage-cartilage biointerface to occur. The precise molecular mechanism through which boundary lubrication is provided by PRG4 and HA in SF at the cartilage surfaces (viscous boundary layer [33], adaptive mechanical control [34]) remains to be fully clarified. However, the results presented here are in general consistent with PRG4 + HA functioning synergistically to reduce friction at a cartilage surface through thick film boundary lubrication as proposed by the adaptive multimodal mechanism [34].

This study used preparations of PRG4 and HA that are representative of their composition within SF. The PRG4 preparation contained both multimeric and monomeric PRG4 species typically found in SF [27], and the R/A preparation was deficient in the multimeric PRG4 which could potentially occur in OA SF. A single high MW HA preparation was used, with 1.5 MDa being within the range of previously reported HA MW distribution in normal and OA SF. [35, 36] Future studies could examine the friction reducing ability of each PRG4 multimeric/monomeric species with HA at a cartilage-cartilage biointerface, as well as an HA solution composed of a mixtures of various MW HA at (patho)physiological concentrations to further examine the potential concentration/MW dependence of the PRG4 + HA synergism. Lastly, while a smaller number of replicates has previously been used to assess differences between lubricants [12], as the lubricating ability of the solutions of interest become more similar in composition and low-friction function, a higher number of replicates may help elucidate if the apparent subtle differences observed here are in fact functionally important.

The coefficients of friction obtained here are consistent with previously measured values for purified solutions of PRG4 and HA, alone and in combination, at a cartilage-cartilage biointerface. <μ_kinetic,Neq_ > for PRG4 at 4.5 and 45 μg/mL observed in previous studies was on the order of 0.2, while PRG4 at 450 μg/mL was 0.10 [5]. μ_static,Neq_ for PRG4 at 450 μg/mL observed in previous studies was on the order of 0.4 [5]. The < μ_kinetic,Neq_ > obtained here for HA at 0.3 mg/mL with PRG4 at 45 and 450 μg/mL (0.152, 0.073) are lower than previously obtained for PRG4 alone, demonstrating friction reduction compared to PRG4 or HA alone even when low concentrations of high MW HA are added to low concentrations of PRG4. <μ_kinetic,Neq_ > for 1.5 MDa HA alone at 3.3 mg/mL was 0.080 in this study, and has been observed to be approximately 0.09 [12]; the values observed here with PRG4 (even 45 μg/mL, 0.072) appear to be similar to 1.5 MDa HA alone, indicating that very low concentrations of PRG4 can limit the boundary lubricating ability of HA + PRG4 solutions. Previous measurements of < μ_kinetic,Neq_ > for 450 μg/mL PRG4 + 3.3 mg/mL 1.5 MDa HA (0.046 [12]) are consistent with the values observed in this study (0.054). Note that μ_static,Neq_ is presented here as a representation of start-up friction, and is calculated from the peak torque measurement at start-up of motion. <μ_kinetic,Neq_ > is calculated from the average torque in the final 2 revolutions of the testing protocol, and is representative of steady-state lubricating ability. Differences is trends between < μ_kinetic,Neq_ > and μ_static,Neq_ (ie. HA at 3.3 mg/mL + PRG4 at 150 μg/mL appears to be equivalent to 45 and 450 μg/mL for μ_static,Neq_ but not for < μ_kinetic,Neq_>) could be due to the fact that there are differences in how friction is reduced in start-up versus steady state motion.

This study provides insight into the effects that PRG4 tertiary and quaternary structure, which may be altered during injury and disease, have on its functional interaction with HA. However, potential changes in PRG4 structure, including relative composition of multimers:monomers and fragments of PRG4 [14], remain to be clarified. Previous preliminary results have demonstrated that < μ_kinetic,Neq_ > of native PRG4 alone at 450 μg/mL is increased 34 % upon R/A, providing evidence that the disulfide-bonded structure of PRG4 itself is important for boundary lubricating ability [16]. In this study, despite a slight reduction in friction, the cartilage boundary lubricating ability of HA alone and HA + R/A PRG4 were not significantly different, suggesting that degradation of PRG4 structure and/or assembly in SF could potentially impact SF boundary lubricating ability by altering the PRG4 + HA interaction. This suggests that PRG4’s tertiary and quaternary protein structure is important in the interaction with HA. Future studies examining the role of PRG4 multimer/monomer interaction with HA to reduce friction will help clarify this issue.

These results also demonstrate that PRG4 can further reduce friction at a cartilage-cartilage biointerface, under boundary mode lubrication, beyond that of a cross-linked HA clinical product alone. Indeed, the < μ_kinetic,Neq_ > obtained for hylan G-F 20 at 3.3 mg/mL and PRG4 at 450 μg/mL (0.048) is very close to those discussed above for PRG4 and 1.5 MDa HA. These results contrast with previous observations using a similar *in vitro* cartilage boundary lubrication test, where it was observed that hylan G-F 20 failed to lubricate as well as SF, and failed to prevent chondrocyte apoptosis compared to SF. [37] Subsequent work demonstrated that addition of purified PRG4 to PRG4-void SF was able to decrease chondrocyte apoptosis, and lower < μ_kinetic,Neq_ > beyond that of PRG4 alone, suggesting again that the PRG4 + HA interaction is critical for normal SF function [38]. While the studies investigating chondrocyte apoptosis and boundary lubrication used a similar *in vitro* boundary lubrication test setup as this study, overall values may differ due to test parameter differences (no annular geometry, less time for stress relaxation, live explants, 12 continuous cycles vs. start and stop). The observation that PRG4 + HA friction reduction is not disrupted by the cross-linking procedure is consistent with previous evidence suggesting that the PRG4 + HA interaction is not a specific site-dependent binding, but rather a physical interaction [12, 39]. The hylan G-F 20 used in this study was diluted to 3.3 mg/mL from its clinical concentration of 8 mg/mL to provide consistency with previous studies characterizing the PRG4 + HA interaction and investigate the effect of cross-linking. The effect of this dilution on PRG4 + HA interaction is currently unclear, and future studies elucidating the mechanism of the PRG4 + HA interaction will provide insight into the effects of supra-physiological HA concentrations and their influence on interaction with PRG4 *in vivo*.

## Conclusion

This study provides further insight into the nature of cartilage boundary lubrication by SF constituents PRG4 and HA. The results presented here demonstrate the importance of both PRG4 and high MW HA concentration, as well as PRG4 and HA structure to their synergistic friction-reducing cartilage boundary lubricating ability. These findings are consistent with observations of cartilage boundary lubrication by SF; when normal HA MW distribution and PRG4 content are decreased, lubricating ability is compromised [13], but when normal HA MW distribution is maintained with low PRG4 concentration, lubricating ability is equivalent to that of normal SF. [40] As cartilage boundary lubrication synergism appears to be lost when both PRG4 and high MW HA are present in low concentrations, it is possible that a combined PRG4 + HA intra-articular treatment may be able to “rescue” SF deficient in either lubricant. Given that combining PRG4 and HA in an intra-articular biotherapeutic treatment may be able to impart the benefits of both HA (pain relief, viscosity) and PRG4 (chondroprotection [23–26], and potentially viscosity [28, 41]), characterizing and understanding the molecular mechanism(s) of the functional synergism could be of great value in optimizing concentrations and/or structural composition to further improve current intra articular biotherapeutic treatments.

## Abbreviations

SF: synovial fluid
PRG4: proteoglycan 4
HA: hyaluronan
PBS: phosphate buffered saline
MW: molecular weight
OA: osteoarthritis
R/A: reduced and alkylated
Neq: equilibrium load
Tps: pre-sliding duration
